# A Nonlinear Fractional Viscoelastic-Plastic Creep Model of Asphalt Mixture

**DOI:** 10.3390/polym13081278

**Published:** 2021-04-14

**Authors:** Yongjun Zhang, Xiu Liu, Boyuan Yin, Wenbo Luo

**Affiliations:** 1 College of Civil Engineering and Mechanics, Xiangtan University, Xiangtan 411105, China; 201731570117@smail.xtu.edu.cn (Y.Z.); liux@xtu.edu.cn (X.L.); 2 Hunan Key Laboratory of Geomechanics and Engineering Safety, Xiangtan University, Xiangtan 411105, China; 3 School of Civil Engineering, Hunan University of Science and Technology, Xiangtan 411201, China; yinboyuanxtu@163.com

**Keywords:** asphalt mixture, nonlinear creep, damage, fractional derivative viscoelasticity, viscoplasticity

## Abstract

The mechanical behavior of asphalt mixture under high stresses presents nonlinear viscoelasticity and permanent deformation. In this paper, a nonlinear fractional viscoelastic plastic (NFVEP) creep model for asphalt mixture is proposed based on the Nishihara model, with a Koeller spring-pot replacing the Newton dashpot. The NFVEP model considers the instantaneous elasticity, viscoelasticity with damage and time-hardening viscoplasticity with damage concurrently, and the viscoelastic response is modeled by fractional derivative viscoelasticity. To verify the model, uniaxial compressive creep tests under various stresses ranging from 0.4 MPa to 0.8 MPa were carried out at room temperature. The NFVEP model predictions are in good agreement with the experiments. The comparison with the modified Nishihara model and the Burgers model reveals the advantages of the NFVEP model. The results show that the NFVEP model, with the same set of parameters, can not only describe the primary and steady-state creep stages of asphalt mixture under low stress levels but also the whole creep process, including the tertiary creep stage, of asphalt mixture under high stress levels.

## 1. Introduction

Hot mixed asphalt (HMA) mixture is one of the most widely used pavement materials and has remarkable viscoelastic properties [1]. Its mechanical behavior exhibits obvious time dependency, temperature dependency and load frequency dependency. Theoretical and experimental studies on its creep, stress relaxation and dynamic mechanical behaviors have provided a crucial foundation for pavement design.

In general, the viscoelasticity of materials can be divided into two categories: linear viscoelasticity and nonlinear viscoelasticity [1,2,3]. It can be distinguished by creep tests at different stress levels. If the creep compliance curve is independent of the applied creep stress, that is, the creep strain is proportional to the creep stress, the material is linearly viscoelastic; otherwise, the material is nonlinearly viscoelastic.

Monismith et al. demonstrated that an asphalt mixture exhibits linear viscoelasticity when the stress level is lower than 0.1 MPa [4]. Subsequently, Airey and Rahimzadeh pointed out that when the deformation of an asphalt mixture is less than 100 microstrains, the asphalt mixture exhibits linear viscoelasticity, and the critical strain limit of linear viscoelasticity varies depending on the modification of the material [5]. Consequently, under intermediate or considerable strains or stresses, its mechanical behavior would be categorized as nonlinear viscoelastic behavior.

The commonly used nonlinear viscoelastic models include multiple integral models, single integral models and power law models [2,3]. Among them, the single integral models have simple forms and have been widely used in engineering applications because of the easy determination of the model kernel function by simple experiments. The Schapery model [6] and its reduced form, the Findley model [7], are the two most recognized single integral constitutive models, and they have been widely used in the nonlinear viscoelastic modeling of composite materials. Gao et al. demonstrated the applicability of the Findley model to describe the nonlinear creep behavior of HMA [8]. In addition, due to fewer parameters and a simpler modeling process, the time-hardening power law model was constructed for nonlinear viscoelastic modeling with the commercial finite element software ABAQUS [9,10]. However, these nonlinear viscoelastic models cannot describe the permanent deformation of HMA creep behavior, as the HMA undergoes plastic deformation at high stresses.

Moreover, different deformation mechanisms exist in the entire creep process of HMA under different stress levels [11]. In the creep process at a lower stress level, the mixture is gradually compacted, resulting in a “consolidation effect”. The aggregates are gradually stacked to construct a skeleton, thus slowing the viscoplastic flow and decreasing the creep rate to a stable value. During creep at higher stress levels, the stress on the aggregate exceeds the frictional resistance between aggregates, thus causing aggregate fragmentation, and destroys the skeleton, consequently accelerating the viscoplastic flow in the tertiary creep stage and creep failure of the material.

To describe the permanent deformation and the three-stage creep process of HMA under high stresses more precisely, one feasible approach is to add a plastic element into the classic integer-order differential viscoelastic models. For instance, with the addition of a plastic element in the Burgers model, the Nishihara model has been widely used in the analysis of viscoelastic and plastic mechanical behaviors of rocks and soils. In the recent decade, some modified Nishihara models were developed to describe the accelerated creep process more precisely [12,13] and have been used in the mechanical analysis of asphalt mixtures [14] and asphalt mortar [15].

Another feasible approach is to couple a continuum damage evolution law with the linear viscoelastic model. Zheng proposed a modified Burgers model to fit the creep compliance master curve by using a damage factor based on the Weibull function [16]. Abu Al-Rub et al. adopted Kachanov’s nondestructive collocation method and used the constitutive model of nondestructive materials to construct a model of damaged materials through the so-called effective stress, and carried out finite element simulation [17]. Grishchenko described the tertiary creep stage of an asphalt mixture with the Kachanov–Rabotnov creep damage model [11]. Zhang et al. considered the competitive mechanism of the strain hardening effect and damage softening effect and proposed an elastic-viscoplastic damage mechanics model to describe the permanent deformation of asphalt mixtures [18]. These works enrich the constitutive theory of asphalt mixtures.

The aim of this paper is to propose a nonlinear viscoelastic-plastic creep model to analyze and describe the creep behavior of asphalt mixtures at different stress levels by taking the viscoelasticity, viscoplasticity and stress-induced damage evolution into consideration. The feature of the model is that the parameters are independent of the creep stress, and the model can predict the behavior of all the creep stages of asphalt mixture at various stress levels.

## 2. Theory

### 2.1. Viscoelastic-Plastic Creep Model Based on Nishihara Model

**Figure 1:**
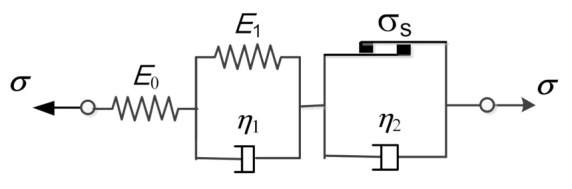
Nishihara model.

Asphalt mixtures are composite materials with elasticity, viscoelasticity and viscoplasticity under high stress, and their mechanical behavior should be described by a viscoelastic-plastic constitutive model. The Nishihara model is a widely used viscoelastic-plastic model that is composed of an elastic element, a Kelvin viscoelastic unit and a viscoplastic unit in series [19], as shown in Figure 1.

The creep equation of the Nishihara model under a constant stress $\sigma_{c}$ can be given as follows:

(1) $$\varepsilon_{c}\left(t\right)=\frac{\sigma_{c}}{E_{0}}+\frac{\sigma_{c}}{E_{1}}\left(1-e^{-t/\tau }\right)+\frac{\langle \sigma_{c}-\sigma_{s}\rangle }{\eta_{2}}t$$

where $\varepsilon_{c}$ is the creep strain, $E_{0}$ and $E_{1}$ are the elastic moduli of the Hooke springs, $\eta_{1}$ and $\eta_{2}$ are the viscosity coefficients of the Newton dashpots, $\tau =\eta_{1}/E_{1}$ is the retardation time of the Kelvin unit, $\sigma_{s}$ is the yield stress of the viscoplastic unit, and $\langle x\rangle =\left(x+\left|x\right|\right)/2$. If $\sigma_{s}=0$, the Nishihara model is reduced to the Burgers model, and the corresponding creep equation is then expressed as:

(2) $$\varepsilon_{c}\left(t\right)=\frac{\sigma_{c}}{E_{0}}+\frac{\sigma_{c}}{E_{1}}\left(1-e^{-t/\tau }\right)+\frac{\sigma_{c}}{\eta_{2}}t$$

The creep failure process of materials usually goes through three stages: the primary creep stage, steady creep stage and tertiary creep stage. The Nishihara model can describe the first two stages well, but it cannot describe the tertiary creep stage as the strain increases linearly with time when the time period becomes great enough. To overcome this shortcoming, many scholars modified the linear time function of the creep behavior of viscoplastic elements into a power law function of time [14,20], which essentially transformed the dashpot element with a constant viscosity coefficient into a dashpot element with a variable viscosity coefficient (=$\eta_{2}t^{1-n}$). Therefore, the creep equation of the modified Nishihara model can be written as:

(3) $$\varepsilon_{c}\left(t\right)=\frac{\sigma_{c}}{E_{0}}+\frac{\sigma_{c}}{E_{1}}\left(1-e^{-t/\tau }\right)+\frac{\langle \sigma_{c}-\sigma_{s}\rangle }{\eta_{2}}t^{n}$$

Although Equation (3) reflects the characteristics of the S-shaped three-stage creep curve, it still describes the linear viscoelastic plastic mechanical behavior, because the creep strain $\varepsilon_{c}\left(t\right)$ is linearly proportional to the applied constant stress $\sigma_{c}$.

### 2.2. Viscoelastic-Plastic Creep Model Based on Fractional Differentiation

**Figure 2:**
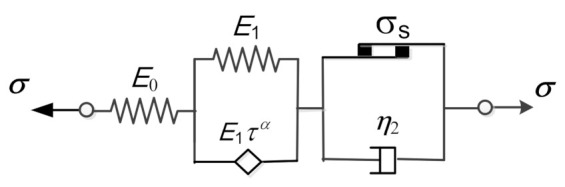
Fractional derivative viscoelastic-plastic model.

The classical integral differential viscoelastic constitutive model characterizes viscoelastic behavior by various combinations of the Hooke spring and Newton dashpot. When the described mechanical behavior is more complex, the model may have many parameters. To overcome this disadvantage, a viscoelastic constitutive theory based on fractional calculus was developed [21,22]. Koeller used a fractional differential operator to define a kind of spring-pot [21], and the stress–strain relation of the spring-pot is written by:

(4) $$\sigma \left(t\right)=E^{1-\alpha }\eta^{\alpha }D^{\alpha }\varepsilon (t)=E\tau^{\alpha }D^{\alpha }\varepsilon (t)$$

where τ=η/E and $D^{\alpha }$ denotes the differential operator of Riemann-Liouville fractional calculus [22], which is defined as:

(5) $$D^{\alpha }f\left(t\right)=\frac{1}{\Gamma \left(1-\alpha \right)}\frac{d}{dt}\int_{0}^{t}{\left(t-\tau \right)}^{-\alpha }f\left(\tau \right)d\tau$$

where α is the fractional order of the differentiation, 0<α<1, $\Gamma \left(\cdot \right)$ is the gamma function, and $\Gamma \left(z\right)=\int_{0}^{\infty }x^{z-1}e^{-x}dx$. According to Equations (4) and (5), the spring-pot reduces to the Hooke spring when α→0, while it reduces to the Newton dashpot when α→1. Thus, the spring-pot can be regarded as a kind of fractional differential viscoelastic element. In this paper, the spring-pot is represented by a diamond, as shown in Figure 2, and we replace the dashpot of the Kelvin unit in Figure 1 with the fractional order spring-pot to obtain the corresponding fractional viscoelastic-plastic model.

Recalling Equation (4) for the spring-pot, the stress–strain relation of the fractional Kelvin element depicted in Figure 2 can be written as:

(6) $$\sigma \left(t\right)=E_{1}\varepsilon_{1}\left(t\right)+E_{1}\tau^{\alpha }D^{\alpha }\left[\varepsilon_{1}\left(t\right)\right]$$

where $\varepsilon_{1}$ is the strain corresponding to the stress σ in the fractional Kelvin element. The Laplace transform to Equation (6) reads:

(7) $$\sigma^{*}\left(s\right)=E_{1}\varepsilon_{1}^{*}\left(s\right)+E_{1}\tau^{\alpha }s^{\alpha }\varepsilon_{1}^{*}\left(s\right)$$

For the case of creep, if $\sigma \left(t\right)=\sigma_{c}H\left(t\right)$ with $H\left(t\right)$ being the Heaviside step function, then $\sigma^{*}\left(s\right)=\sigma_{c}/s$, and $\varepsilon_{1}{}^{*}\left(s\right)$ is then written as:

(8) $$\varepsilon_{1}{}^{*}\left(s\right)=\frac{\sigma_{c}}{E_{1}\left(s+\tau^{\alpha }s^{\alpha +1}\right)}$$

The creep strain of the fractional-order Kelvin element can be obtained by the inverse Laplace transform to Equation (8) and expressed as below:

(9) $$\varepsilon_{c}\left(t\right)=\frac{\sigma_{c}}{E_{1}}\left\{1-E_{\alpha ,1}\left[-{\left(t/\tau \right)}^{\alpha }\right]\right\}$$

where $E_{\alpha ,1}\left(z\right)=\sum_{k=0}^{\infty }z^{k}/\Gamma \left(\alpha k+1\right)$ for α>0, which is the so-called single parameter Mittag–Leffler function, and $E_{1,1}\left(z\right)=e^{z}$ when α = 1. Thus, when α=1, $\varepsilon_{c}\left(t\right)=\sigma_{c}\left(1-e^{-t/\tau }\right)/E_{1}$; consequently, the fractional Kelvin element becomes the classical Kelvin element in such case. Therefore, similarly to Equation (3), the creep equation of the fractional viscoelastic-plastic model shown in Figure 2 can be written as:

(10) $$\varepsilon_{c}\left(t\right)=\frac{\sigma_{c}}{E_{0}}+\frac{\sigma_{c}}{E_{1}}\left\{1-E_{\alpha ,1}\left[-{\left(t/\tau \right)}^{\alpha }\right]\right\}+\frac{\langle \sigma_{c}-\sigma_{s}\rangle }{\eta_{2}}t^{n}$$

It should be noted that the creep strain $\varepsilon_{c}\left(t\right)$ in Equation (10) is linearly proportional to the constant stress $\sigma_{c}$, as it does in Equation (3). Thus Equation (10) describes only the linear viscoelastic-plastic creep.

### 2.3. Nonlinear Fractional Viscoelastic-Plastic Creep Model Considering Damage Effect

Damage evolution under loading will result in nonlinear mechanical behavior. When subjected to higher stresses, the viscoelastic-plastic damage with cavitation and microcracking as the main characteristics will occur in the asphalt mixture and cause accelerated creep until failure.

Let ω represent the damage variable, and its evolution is related to the stress level as well as its own damage state, that is, $d\omega /dt=f\left(\sigma ,\omega \right)$. Rabotnov [23] and Leckie et al. [24] proposed a power law damage evolution rule, which is defined as:

(11) $$d\omega /dt=c\sigma^{m}{\left(1-\omega \right)}^{-q}$$

where σ is the nominal stress and *c*, *m* and *q* are the temperature-dependent material constants. Considering the initial condition of $\omega =0^{+}$ at $t=t_{0}$ and the critical condition of ω=1 at $t=t_{r}$, the damage evolution equation can be obtained by integrating Equation (11):

(12) $$\omega =1-{\left[1-\left(1+q\right)\int_{t_{0}}^{t}c\sigma^{m}dt\right]}^{\frac{1}{1+q}},\;\left(t_{0}\leq t\leq t_{r}\right)$$

where *t*_0_ is the damage initiation time and *t_r_* is the damage-induced failure time. For the case of creep, $\sigma \left(t\right)=\sigma_{c}H\left(t\right)$, if the damage occurs at the moment of loading, that is, *t*_0_ = 0, then the evolution equation of creep damage can be written as:

(13) $$\omega =1-{\left(1-\frac{t}{t_{r}}\right)}^{\frac{1}{1+q}},t_{r}=\frac{1}{c\left(1+q\right)\sigma_{c}^{m}}$$

where *t_r_* represents the creep rupture time for the given temperature and creep stress level.

According to the principle of strain equivalence of continuum damage mechanics [25], the constitutive relationship of damaged material is consistent with that of a fictitious undamaged state, said an effective continuum, and it is only necessary to replace the nominal stress with the effective stress. The relationship between the effective stress $\bar{\sigma }$ and the nominal stress σ is expressed as:

(14) $$\bar{\sigma }=\frac{\sigma }{1-\omega }$$

Considering Equations (10), (13) and (14), the nonlinear fractional viscoelastic-plastic (NFVEP) creep model considering damage evolution can be written as:

(15) $$\varepsilon_{c}\left(t\right)=\frac{\sigma_{c}}{E_{0}}+\frac{{\bar{\sigma }}_{c}}{E_{1}}\left\{1-E_{\alpha ,1}\left[-{\left(t/\tau \right)}^{\alpha }\right]\right\}+\frac{\langle {\bar{\sigma }}_{c}-\sigma_{s}\rangle }{\eta_{2}}t^{n}$$

where *E*_0_, *E*_1_, *α*, *τ*, *η*_2_, *n*, *q*, *c* and *m* are temperature-related material parameters and determined by test data fitting. Equation (15) is not convenient for engineering applications because of the computational complexity of the involved Mittag-Leffler function. Let *α* = 1 in the gamma function $\Gamma \left(k\alpha +1\right)$, as Yin et al. did in their work [26], then, $E_{\alpha ,1}\left[-{\left(t/\tau \right)}^{\alpha }\right]$ can be simplified as $\exp\left[-{\left(t/\tau \right)}^{\alpha }\right]$, consequently, Equation (15) can be simplified as:

(16) $$\varepsilon_{c}\left(t\right)=\frac{\sigma_{c}}{E_{0}}+\frac{{\bar{\sigma }}_{c}}{E_{1}}\left\{1-\exp\left[-{\left(t/\tau \right)}^{\alpha }\right]\right\}+\frac{\langle {\bar{\sigma }}_{c}-\sigma_{s}\rangle }{\eta_{2}}t^{n}$$

Combining Equations (13), (14) and (16) shows the simplified NFVEP creep model.

## 3. Compressive Creep Tests

### 3.1. Material and Methodology

According to the Chinese standard JTG D50-2017 [27], the standard axle load for pavement design is 100 kN, and the corresponding tire grounding pressure is 0.7 MPa; in real cases the axle loads of most trucks range from 60 kN to 120 kN, and the corresponding tire grounding pressure varies from 0.4 MPa to 0.8 MPa. In order to verify the NFVEP model proposed in the previous section, compressive creep tests on asphalt mixture specimens were conducted at various stress levels and at room temperature. The applied stresses were selected to range from 0.4 MPa to 0.8 MPa in accordance with the above-mentioned considerations.

Zhonghai AH-70 heavy-duty road asphalt (produced by Zhonghai Bitumen Co. Ltd., Binzhou, China) was selected to prepare the AC-13C asphalt mixture specimens, and the main quality indices of the asphalt were measured and listed in Table 1. The asphalt mixture gradation was designed to meet the Chinese standard JTG F40-2004 [28], which is listed in Table 2, and the asphalt-aggregate mass ratio was determined to be 4.6% in accordance with JTG F40-2004. The AC-13C asphalt mixture cylindrical specimens with a diameter of 100 mm and a height of 150 mm were used for uniaxial compressive creep tests. The creep tests were carried out at 26 °C on the MTS809 test machine (MTS Systems Corporation, Eden Prairie, MN, USA), and the applied constant compressive stresses were 0.4 MPa, 0.5 MPa, 0.6 MPa, 0.7 MPa and 0.8 MPa, respectively. The tests at every stress level were repeated at least four times and each specimen was tested only once. To reduce the influence of friction between the clamps and the specimen, a double-layer polytetrafluoroethylene (PTFE) film was placed on both ends of the specimen.

### 3.2. Test Results

**Figure 3:**
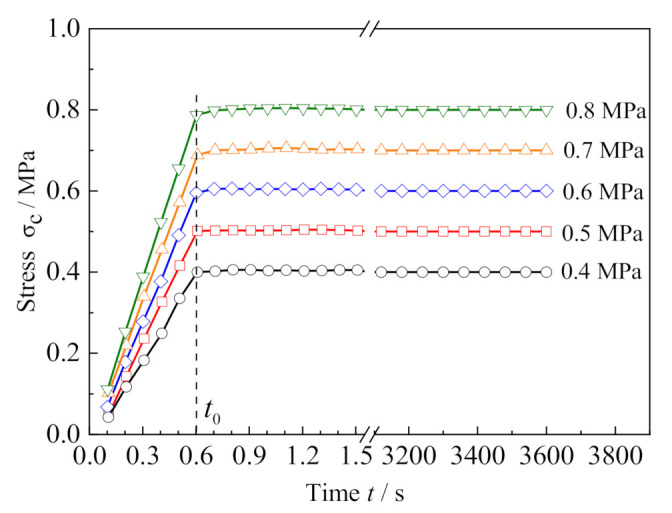
Loading histories of creep tests.

**Figure 4:**
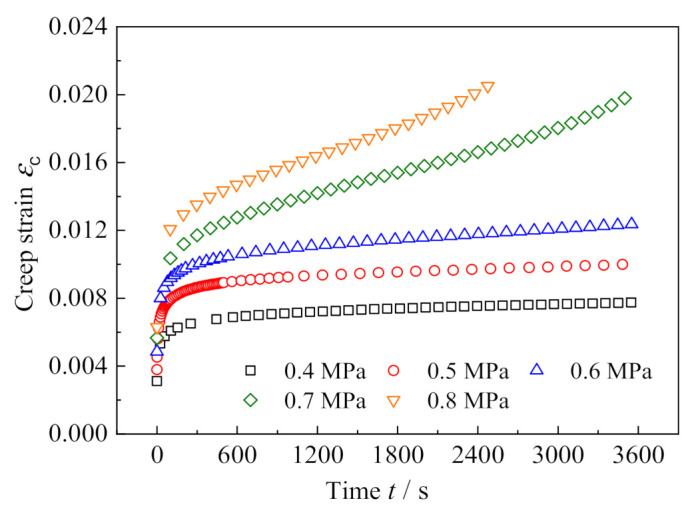
Creep strain vs. time curves at various stresses.

Due to the limitation of the loading equipment and measurement instruments, it is difficult to reach the given stress level instantaneously, and the creep test requires a ramp loading period so that the applied stress can reach the set value in the shortest possible time. In this paper, all the preset stresses were loaded in 0.6 s and maintained for 1 h. Figure 3 shows the loading histories of the creep tests and the corresponding creep strain vs. time curves for the five applied stresses are presented in Figure 4. The curves indicate that when the stress level is relatively low (e.g., σ_c_ = 0.4 MPa, 0.5 MPa and 0.6 MPa), the creep presents only two stages: the primary creep stage and the steady-state creep stage. However, when the stress level increases to 0.7 MPa and 0.8 MPa, the tertiary creep stage occurs.

## 4. Analysis and Discussion

**Figure 5:**
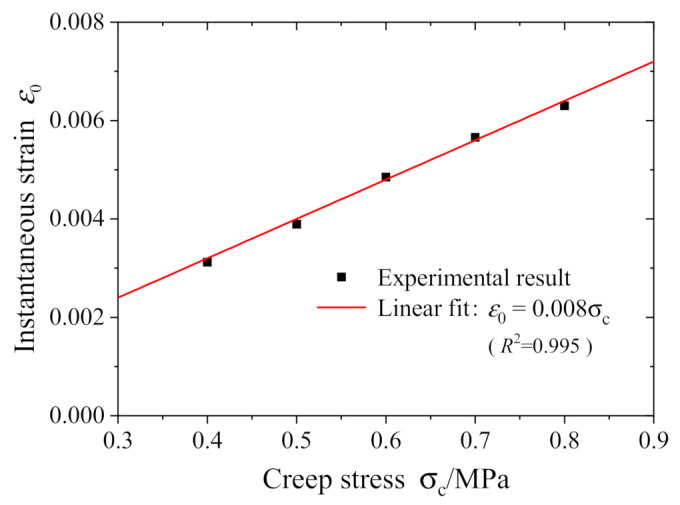
Initial instantaneous strain for various creep stresses.

In this section, we check out whether the NFVEP model can accurately describe and reproduce all the creep behaviors as presented in the tests. As the ramp loading time in the tests is much shorter than the creep duration, the strains at the end of ramp loading, i.e., at 0.6 s, are regarded as the initial instantaneous strain in creep tests. Figure 5 shows the test results of the initial instantaneous strain for five different creep stresses. It is obvious that the initial instantaneous strain increases linearly with the applied stress, satisfying the linear elastic relationship. Linear fitting Equation (16) at *t* = 0 with the data in Figure 5 determines the elastic modulus *E*_0_ (=125 MPa).

**Figure 6:**
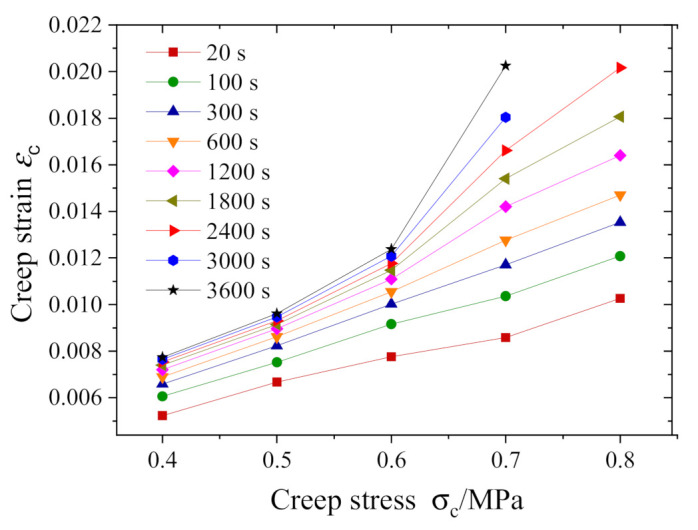
Isochronous stress–strain curves.

In order to identify whether the creep is nonlinear, as shown in Figure 6, we extracted data pairs of creep stress and strain at nine different moments from Figure 4 to plot the isochronous stress–strain curves. Clearly, when the creep time exceeds 600 s, the isochronous stress–strain curves reflect a nonlinear relationship; moreover, the longer the creep time is, the more significant the nonlinearity is. Since the curve slope at each data point in Figure 6 represents the creep compliance under the specified creep stress, the nonlinear isochronous stress–strain curve indicates that the creep compliance is stress-dependent. Such a feature proves the nonlinear creep.

**Figure 7:**
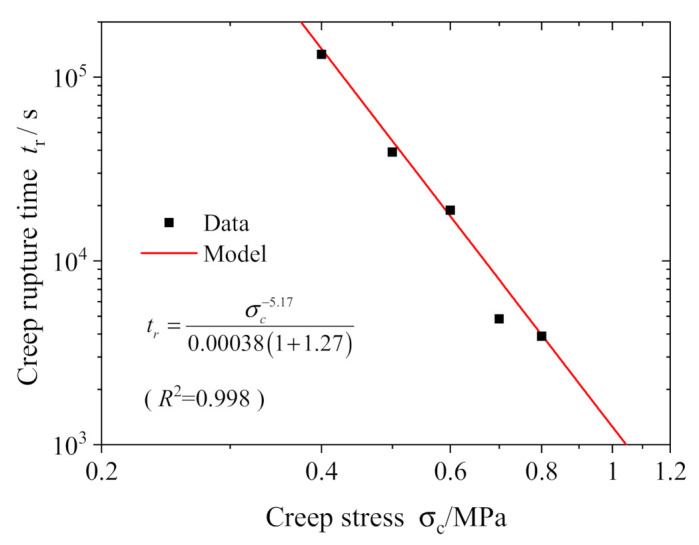
Variation of *t*_r_ with creep stress.

Gonzalez et al. conducted a large number of tests on asphalt mixture and determined that the yield stress *σ*_s_ at various temperatures was 0.04~0.06 MPa [29]. We set $\sigma_{s}=0.04$ MPa in this work for the later analysis. The other model parameters in Equation (16) are determined by curve fitting to the creep test data with the Levenberg–Marquardt nonlinear least squares algorithm. The quality of fit is evaluated by the *R*^2^ value, $R^{2}=1-SSE/SST$, where *SSE* is the residual sum of squares between the calculated values of the model and the experimental data and *SST* is the total sum of squares of the deviations. Table 3 lists the determined model parameters *E*_1_, *α*, *τ*, *η*_2_, *n*, *q* and *t_r_*. It is seen that *t_r_* decays with creep stress in a power law, as shown in Figure 7 for more confidence; then, the model parameters *c* and *m* are eventually obtained by nonlinear fitting of Equation (13) with the data in Figure 7, and as also listed in Table 3, the quality of fit is *R*^2^ = 0.998.

**Figure 8:**
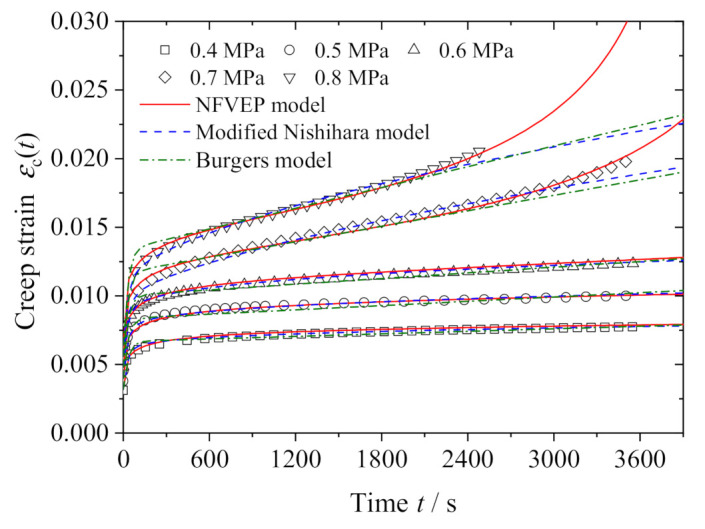
Model predictions vs. test data for different stresses.

**Figure 9:**
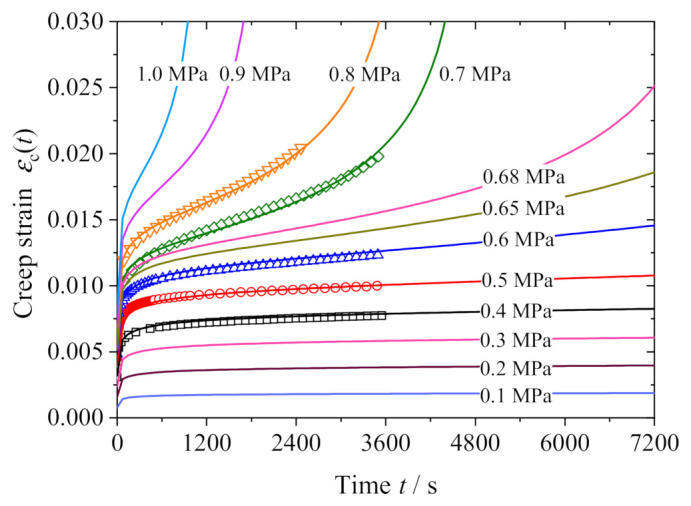
NFVEP model predictions at various stresses.

Figure 8 shows the comparison between the NFVEP model prediction and the test data. In addition, the predictions from the Burgers model (Equation (2)) with parameters in Table 4 and the modified Nishihara model (Equation (3)) with parameters in Table 5 are also presented in the same figure. It is shown that the Burgers model overpredicts the strain in the primary creep stage; neither the Burgers model nor the modified Nishihara model can reproduce the tertiary creep stage at high stresses, and the NFVEP model coincides with the test data in all the three creep stages; in particular, the NFVEP model provides a good description of the tertiary creep stage. To further illustrate this feature, we predict the creep strains at various stresses ranging from 0.1 MPa to 1.0 MPa, including the applied stresses in creep tests. Figure 9 shows the model predictions. It is consistent with the experimental observation that the creep only presents the first two stages when the applied stress is less than 0.6 MPa, indicating the consolidation effect in the asphalt mixture, while the creep under stresses greater than 0.6 MPa includes the tertiary stage. The tertiary creep may be caused by the destruction of the aggregate skeleton under high stress. It is worth noting that all the NFVEP model predictions are based on the same set of parameters as listed in Table 3.

## 5. Conclusions

(a) The compressive creep tests show that the creep behavior of the asphalt mixture exhibits consolidation effect at low stresses and tertiary stage at high stresses. The nonlinearity of the isochronous stress–strain curves reflects the nonlinear characteristics of the creep of the asphalt mixture, and the creep becomes more significantly nonlinear with increasing damage in the tertiary creep stage.

(b) A nonlinear fractional viscoelastic-plastic creep model is proposed by replacing the Newton dashpot in the Nishihara model with the Koeller spring-pot. It considers instantaneous elasticity, fractional viscoelasticity with damage and time-hardening viscoplasticity with damage concurrently, and is applicable to describe the entire creep process, including the tertiary creep stage, of an asphalt mixture under various stresses using the same set of model parameters.

## Figures and Tables

**Table 1:** Quality indices of Zhonghai AH-70 heavy-duty road asphalt.

Penetration Degree (25 °C, 100 g, 5 s) (0.1 mm)	Softening Point (Ball-Circling Method) (°C)	Ductility (15 °C, 5 cm/min) (cm)	Density (25 °C) (kg/m^3^)
68	47.5	>150	1030

**Table 2:** Gradation of AC-13 asphalt mixture.

	Passing Rate/%									
	(Sieve Size/mm)									
	16	13.2	9.5	4.75	2.36	1.18	0.6	0.3	0.15	0.075
Upper limit	100	100.0	85.0	68.0	50.0	38.0	28.0	20.0	15.0	8.0
Lower limit	100	90.0	68.0	38.0	24.0	15.0	10.0	7.0	5.0	4.0
Screening result	100	93	74	51	35	24	17	12	8	5

**Table 3:** The nonlinear fractional viscoelastic plastic (NFVEP) creep model parameters.

*E*_0_/MPa	*E*_1_/MPa	*τ*/s	*α*	*η*_2_/(MPa·s)	*n*	*q*	*c*	*m*
125	128	46	0.38	2000	0.26	1.27	0.00038	5.17
***σ*_c_/MPa**			***t*_r_/s**			***R*^2^**		
0.4			133,000			0.956		
0.5			39,103			0.968		
0.6			18,868			0.983		
0.7			4833			0.989		
0.8			3889			0.997		

**Table 4:** The Burgers model parameters.

*σ_c_*/MPa	*E*_0_/MPa	*E*_1_/MPa	*τ*/s	*η*_2_/(MPa·s)	*R* ^2^
0.4 MPa	125	115	32	1168624.77	0.921
0.5 MPa	125	115	32	879915.62	0.935
0.6 MPa	125	115	32	811050.46	0.939
0.7 MPa	125	115	32	351050.70	0.968
0.8 MPa	125	115	32	301207.26	0.972

**Table 5:** The modified Nishihara model parameters.

*σ_c_*/MPa	*E*_0_/MPa	*E*_1_/MPa	*τ*/s	*η*_2_/(MPa·s)	*n*	*R* ^2^
0.4 MPa	125	185	32	986.79	0.23	0.979
0.5 MPa	125	185	32	1126.12	0.26	0.977
0.6 MPa	125	185	32	1600.82	0.31	0.989
0.7 MPa	125	185	32	13119.85	0.64	0.985
0.8 MPa	125	185	32	9161.37	0.60	0.985

## Data Availability

The data presented in this study are available on request from the corresponding author.
